# Dynamic Arterial Elastance to Predict Mean Arterial Pressure Decrease after Reduction of Vasopressor in Septic Shock Patients

**DOI:** 10.3390/life13010028

**Published:** 2022-12-22

**Authors:** Paolo Persona, Tommaso Tonetti, Ilaria Valeri, Emanuele Pivetta, Francesco Zarantonello, Tommaso Pettenuzzo, Alessandro De Cassai, Paolo Navalesi

**Affiliations:** 1 Institute of Anaesthesia and Intensive Care, Padua University Hospital, 35128 Padua, Italy; 2 Department of Medical and Surgical Sciences (DIMEC), Alma Mater Studiorum—University of Bologna, 40126 Bologna, Italy; 3 Anesthesiology and Intensive Care Medicine, IRCCS Azienda Ospedaliero-Universitaria di Bologna, Policlinico di S. Orsola, 40138 Bologna, Italy; 4 Division of Emergency Medicine and High Dependency Unit, AOU Città della Salute e della Scienza di Torino, Department of Medical Sciences, University of Turin, 10124 Turin, Italy; 5 Department of Medicine (DIMED), University of Padua, Via Vincenzo Gallucci 13, 35121 Padua, Italy

**Keywords:** dynamic elastance, PPV/SVV, septic shock, haemodynamic monitoring

## Abstract

After fluid status optimization, norepinephrine infusion represents the cornerstone of septic shock treatment. De-escalation of vasopressors should be considered with caution, as hypotension increases the risk of mortality. In this prospective observational study including 42 patients, we assess the role of dynamic elastance (EaDyn), i.e., the ratio between pulse pressure variation and stroke volume variation, which can be measured noninvasively by the MostCare monitoring system, to predict a mean arterial pressure (MAP) drop > 10% 30 min after norepinephrine reduction. Patients were divided into responders (MAP falling > 10%) and non-responders (MAP falling < 10%). The receiver-operating-characteristic curve identified an area under the curve of the EaDyn value to predict a MAP decrease > 10% of 0.84. An EaDyn cut-off of 0.84 predicted a MAP drop > 10% with a sensitivity of 0.71 and a specificity of 0.89. In a multivariate logistic regression, EaDyn was significantly and independently associated with MAP decrease (OR 0.001, 95% confidence interval 0.00001–0.081, *p* < 0.001). The nomogram model for the probability of MAP decrease > 10% showed a C-index of 0.90. In conclusion, in a septic shock cohort, EaDyn correlates well with the risk of decrease of MAP > 10% after norepinephrine reduction.

## 1. Introduction

Septic shock management mainly relies on two treatments: source control and organ support. Source control is usually accomplished via surgical approach, when feasible, and antibiotic therapy. Organ support serves as a bridge correcting physiologic derangements, such as organ hypoperfusion, while source control has time to act.

The current clinical practice is based on the available guidelines, that recommend norepinephrine as the treatment of choice for hypotension in septic shock, titrated to maintain an adequate mean arterial pressure (MAP) and to minimize fluid overload [1]. Titration of vasopressor to a target MAP of 65 mmHg is recommended to maintain adequate oxygen delivery and consumption, enhance microcirculatory function and optimize renal function [2].

Once haemodynamic stability has been achieved, early de-escalation of vasopressor therapy should be considered in order to avoid negative side effects, such as tissue hypoperfusion due to excessive vasoconstriction or arrhythmias due to β1-agonist effect [3]. Moreover, recent evidence suggests harm in patients that received high doses of vasopressor agents [4], underlining the importance of frequent patient status re-evaluation and early weaning from vasopressors. On the other hand, reducing norepinephrine too early could be harmful as well, resulting in ongoing organ hypoperfusion: the time spent under the threshold of 65 mmHg is associated with acute kidney injury, myocardial infarction and increase in mortality [5,6]. Although norepinephrine is extensively recognized as the first choice as vasopressor agent in septic shock [7,8], when and how to reduce and stop its infusion is still a matter of debate. Indeed, only a few studies report data on norepinephrine dose reduction strategy [9]. Some authors have suggested a de-escalation strategy based on the value of dynamic arterial elastance (EaDyn), i.e., the ratio of pulse pressure variation (PPV) over stroke volume variation (SVV): according to these studies, EaDyn could potentially act as a dynamic indicator of arterial tone, useful to enhance a functional approach to vasoactive therapy management [10,11,12]. If SVV overweighs PPV this suggests the presence of high arterial tree compliance, thus reduction in norepinephrine dose is probably not beneficial. Unfortunately, the data reported in other studies originate mainly from a limited number of patients, mostly suffering from vasoplegic syndrome secondary to cardiopulmonary bypass (CPB) [11,12,13]. 

PPV and SVV can be measured using several strategies [14]. A haemodynamic monitoring system based on pulse contour analysis, measuring PPV and SVV from invasive arterial pressure waveform, appears to be a priori an ideal tool to measure EaDyn. Differently from those devices assessing PPV and SVV from the same algorithm [15], the pressure recording analytical method (PRAM) used by MostCare (Vytech Health™, Vygon, Padova, Italy) allows the measurement of PPV and SVV avoiding mathematical coupling by applying two separate algorithms.

The aim of our study is to investigate whether EaDyn, measured by minimally invasive haemodynamic monitor MostCare, is able to predict a decrease in MAP of more than 10% after reduction of norepinephrine dose in a cohort of septic-shock patients.

## 2. Materials and Methods

This prospective observational study (approval by Padova University Ethical Committee protocol number 26658) was conducted in the intensive care unit (ICU) of Padua University Hospital, enrolling all consecutive septic-shock patients admitted between July 2018 and July 2019.

Inclusion criteria were: age > 18 years old, septic shock (based on definitions by Evans et al. [1]: Septic shock is a subset of sepsis with circulatory and cellular/metabolic dysfunction associated with a higher risk of mortality), ongoing infusion of norepinephrine and invasive controlled mechanical ventilation with tidal volume > 7 mL/Kg of predicted body weight (PBW). Exclusion criteria were: arrhythmias, resonance artefacts on arterial waveform (excluded by offline fast-flush test performed offline [16]), and concomitant therapy with epinephrine, dobutamine or dopamine.

Haemodynamic parameters were collected by continuous pulse wave analysis performed by MostCare monitor. MostCare estimates stroke volume (SV) from arterial waveform based on the formula SV = A/Z, where A is the area of the systolic phase and Z is the impedance of the system. Z is calculated as P/t × K, where P is the instant pressure and t is time, considering a beat-to-beat analysis of the whole cardiac cycle and the waves reflected from the arterial system. K is calculated as the ratio between expected and measured MAP by assuming constant expected MAP according to Guyton studies [17].

Heart rate (HR), systolic (SP), diastolic (DP), dicrotic (DIP) and mean (MAP) arterial pressure, SV, cardiac output (CO), cardiac index (CI), systemic vascular resistances (SVR), PPV, SVV, EaDyn, and arterial elastance (calculated as DIP over SV) were continuously recorded.

In common with all pulse contour techniques, even MostCare is highly dependent on correct pressure signal, with the risk of reporting unreliable data in cases of resonance artefacts. In order to exclude artefacts we performed a fast-flush test on arterial signal waveform before patient enrolment. In case of uncertain results, an offline analysis based on natural frequency, damping coefficient and amplitude ratio was performed, as previously described [16].

Norepinephrine dose was decreased by 0.03 mcg/Kg/min steps according to our protocol, when MAP was greater than65 mmHg.

**Figure 1:**
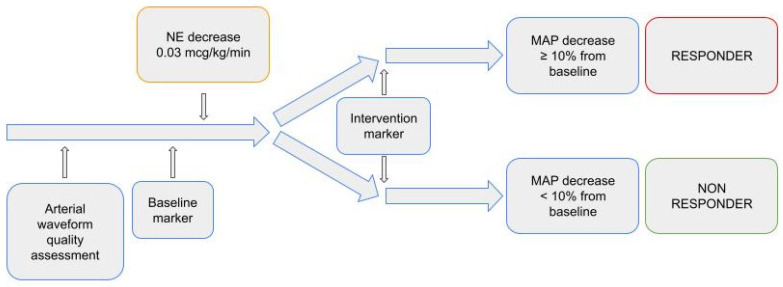
Study diagram.

On MostCare monitoring system, we set a baseline marker before norepinephrine dose reduction (T0) and another 30 min after (T1). Ventilator settings and sedation were left unchanged throughout the observational period. The diagram of the study is depicted in Figure 1.

We calculated that to demonstrate that EaDyn could predict a reduction in MAP after decrease in norepinephrine infusion, with a power of 80%, an area under the receiver-operating-characteristic (ROC) curve (AUC) greater than 0.80 and a risk of 0.05, a sample of at least 30 patients is needed.

Continuous variables are expressed as mean ± standard deviation (SD) or median and interquartile range, according to the data distribution assessed through Shapiro–Wilk test and compared with Student’s *t* test and the Kruskal–Wallis test, as appropriate. Categorical variables are expressed as number and percentage and compared using the χ2 test and the Fisher exact test, as appropriate. The study population was divided into responders (R) and non-responders (NR), based on whether MAP decreased more or less than 10% after 30 min from dose reduction, respectively. Patient characteristics between the two groups and variables before and after dose reduction are compared. Univariate logistic models were used to assess the association between EaDyn and several independent variables. Subsequently, a multivariable analysis was performed by including those variables considered as clinically significant. A Spearman ρ2 analysis and a nomogram model based on the multivariate logistic regression were used with an internal calibration at 1000 repetition boot. The model concordance index (C-index) was calculated to verify the accuracy of the prognostic model. Statistical significance was set at *p* < 0.05. The analysis was performed using the STATA 13 software (Stata Statistical Software, College Station, TX, USA, StataCorp LP), GraphPad Prism 8.1.2 (GraphPad Software, San Diego, CA, USA) and R Statistical Software (R Core Team, R Foundation for Statistical Computing, Vienna, Austria).

## 3. Results

Fifty consecutive septic-shock patients were enrolled. After exclusion of eight patients because of resonance artefacts before or after norepinephrine dose reduction, 42 patients were analysed. Patient anthropometric characteristics and clinical information are shown in Table 1. Haemodynamic parameters before and after norepinephrine decrease in the two groups are shown in Table 2. The median time (hours) from ICU admission to start the weaning from norepinephrine therapy was 38 h (22–46). The median time (hours) from ICU admission to start the weaning from norepinephrine therapy was 38 h (22–46). Fluid responsive patients received a median of 2840 mL/die (2120–3450) of crystalloid or colloid solutions during the resuscitation phase.

In our cohort, n = 18 (43%) patients were responders and n = 24 (57%) non-responders. In responders, MAP changed from a median value of 72 (71–85) mmHg to 64 (63–74) mmHg (*p* < 0.005), while PPV increased from 6 (4–7) to 9 (8–10) median value (*p* < 0.005). In both responders and non-responders, norepinephrine dose decreased from 0.14 to 0.1 mcg/Kg/min (*p* < 0.005). The other variables did not significantly change after norepinephrine dose reduction. The differences inter- and intra-groups are not statistically significant if not displayed. 

**Figure 2:**
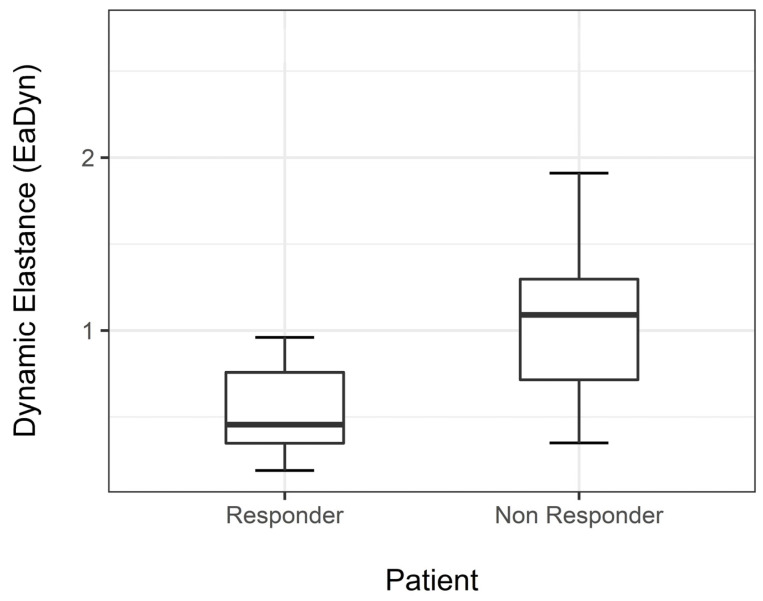
Median EaDyn before drug reduction in non-responders and responders.

Median EaDyn before drug dose reduction was significantly different between R and NR (*p* < 0.001) (Figure 2).

**Figure 3:**
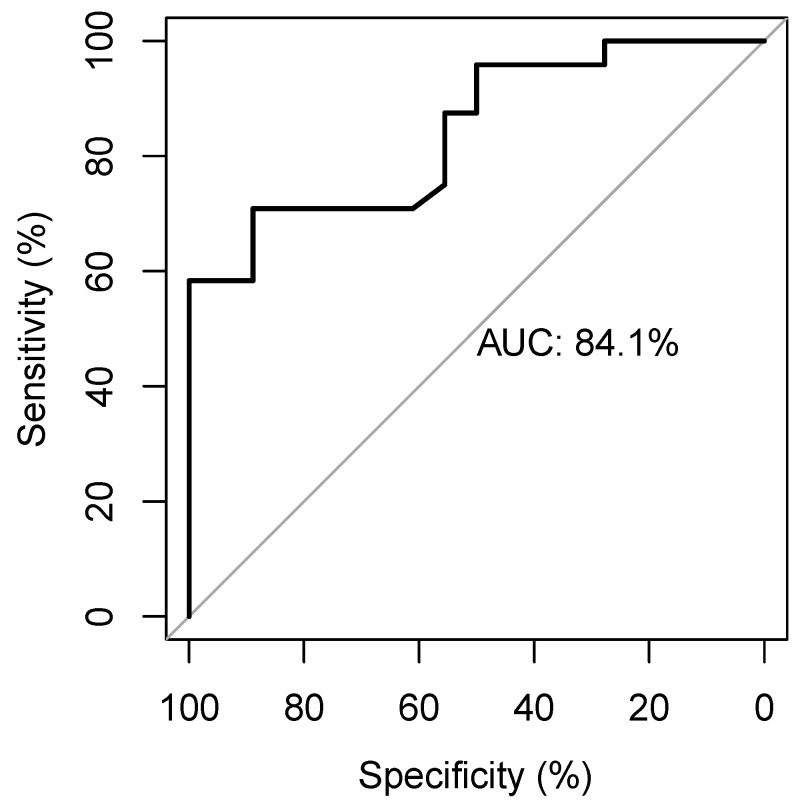
The receiver-operating-characteristic curve showing the diagnostic accuracy of the EaDyn for predicting MAP change.

The ROC curve identifies an AUC of 0.84 and a cut-off of 0.84 with a sensitivity of 0.71 and a specificity of 0.89 (Figure 3) to predict MAP change.

**Figure 4:**
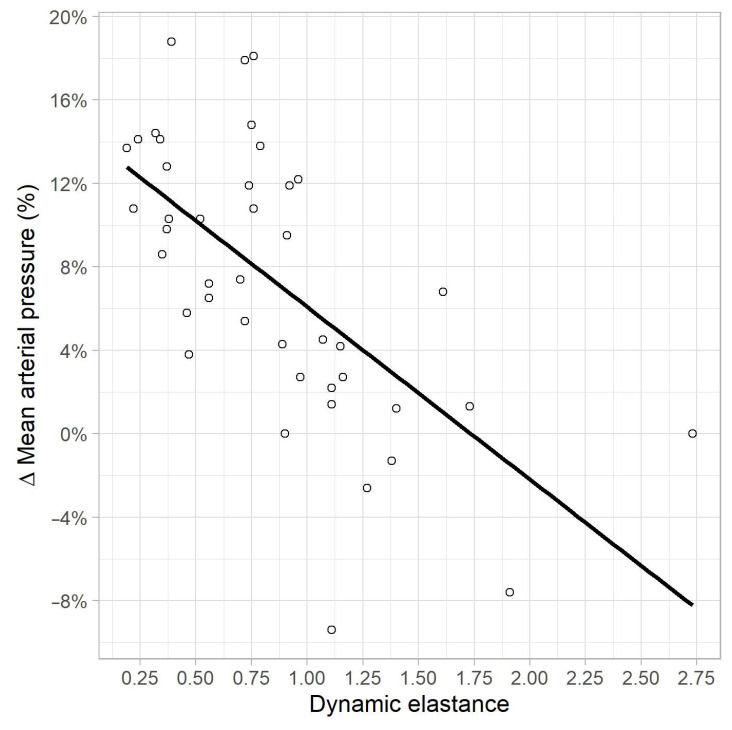
Correlation between MAP variation (%) and EaDyn pre-variation.

In the univariate linear regression analysis, a correlation was found between MAP variation, and EaDyn pre-variation (R^2^ = 0.41, *p* < 0.01) (Figure 4), PPV pre-variation (R^2^ = 0.13, *p* = 0.015) and Ea pre-variation (R^2^ = 0.09, *p* = 0.04). No correlations were found between MAP variation, and pre-variation values of MAP (*p* = 0.46), SVRI (*p* = 0.10), CI (*p* = 0.3), SVV (*p* = 0.08) and MAP-dicrotic pressure (*p* = 0.65).

**Figure 5:**
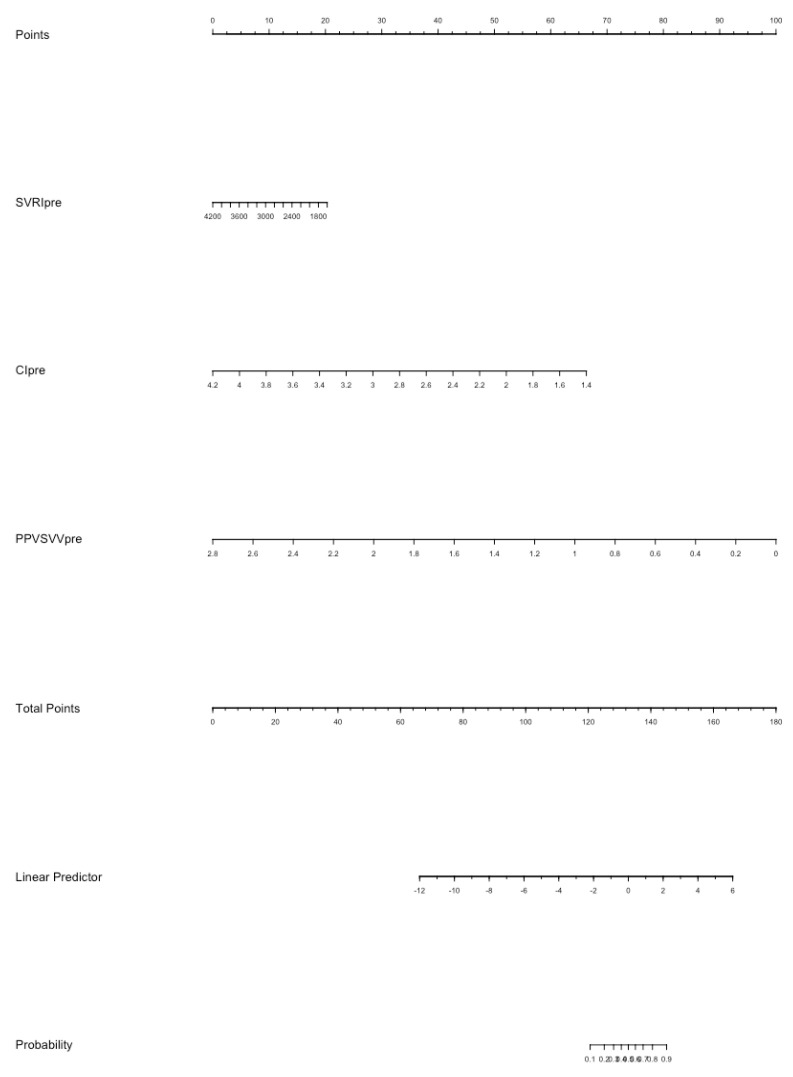
The nomogram model for the probability of MAP decrease after reduction of norepinephrine dosage. Each value of each variable corresponds to some points in the line ‘Total points’; the sum of all points together corresponds to a probability from 0.1 to 0.9 of MAP decrease in the line ‘Probability’.

In the multivariate logistic analysis (Table S1), CI (*p* = 0.026) and EaDyn (*p* = 0.002) show a significant correlation with MAP decrease. The nomogram model for the probability of MAP decrease was built using SVRI, CI, Ea and EaDyn (Figure 5); the internal validation of the model performed at 1000 repetitions boot is depicted in Figure S1; the C-index was 0.897.

## 4. Discussion

In this prospective observational study including 42 adult patients with septic shock undergoing norepinephrine dose reduction, we found that EaDyn, as measured by the minimally invasive monitoring system MostCare, was accurate in predicting a MAP drop greater than 10% after 30 min and was independently associated with MAP decrease.

In clinical practice, the usual approach to vasopressor reduction is based on trial and error, i.e., if vasopressor reduction results in a minimal decrease in MAP, then the de-escalation is considered successful [18], if instead a marked drop in MAP occurs, vasopressor is increased back to the previous level or higher, until a new haemodynamic stability is achieved. It is noteworthy that the risk of developing acute kidney injury or myocardial injury is associated with the time spent under a defined threshold of MAP [19]. Therefore, the identification of a parameter that could predict the change in vascular tone after variations in vasopressor dose would be of great interest. Guinot et al. investigated the response of arterial tone to reduction of vasopressor dose in septic patients but, differently from our study, they measured EaDyn by transpulmonary thermodilution [11], which is a more invasive method to assess haemodynamic parameters: a central venous line and a femoral arterial catheter with the proper thermistor are needed, and repeated calibrations with cold saline boluses are essential to obtain correct estimations. Moreover, PPV and SVV are not measured beat by beat, as the measurement is the average over 30 s [20]. Bar et al. considered the same outcome in a generic vasoplegic population using an uncalibrated pulse contour analysis system to obtain the SVV value, while PPV value was derived from a different multiparametric monitor and the two values were computed offline, due to mathematical coupling of PPV with SVV in the haemodynamic monitoring system of choice [15]. To the best of our knowledge, the MostCare system is currently the only haemodynamic monitoring system computing PPV/SVV in real time; PPV and SVV are estimated by different algorithms and the measurement is only affected by arrhythmias and resonance artefacts of arterial waveform [21]. The fact that a single haemodynamic monitoring system measures both PPV and SVV does not mean that the two variables are mathematically coupled [22]. The opportunity to obtain continuous data on arterial tone of septic patients could be the starting point to a tailored and more rapid de-escalation of vasopressor agents like norepinephrine. If pulse pressure increases more than stroke volume in response to a variation of intrathoracic pressure related to the respiratory cycle, the arterial tone may be high and the system not compliant with the variation of volume. In this case, the decrease of norepinephrine dose could minimally affect the arterial tone without any significant impact on MAP [13]. Based on these considerations, EaDyn was also evaluated as a predictor of positive effects of volume expansion [23,24]; on the other hand, many studies have evaluated different tests to predict fluid responsiveness [25,26]; nevertheless, modalities and timing of the reduction in vasopressor dose are still far from being guided by a defined parameter. We found a weak linear correlation between EaDyn and MAP before norepinephrine dose reduction (R^2^ = 0.41). This correlation was stronger than the one reported in a previous study [11]. This suggests that EaDyn might help to predict MAP reduction after norepinephrine titration, thus avoiding the risk of overcoming the dangerous threshold. 

An EaDyn cut-off of 0.84 was found to discriminate responders (MAP falling > 10%) from non-responders (MAP falling < 10%). Other authors found a higher cut-off value. However, the study populations are not comparable due to the causes of vasoplegia [12] and basal haemodynamic conditions [11]. The underlying disease, the adrenergic receptors status and other factors are responsible for the high variability in response to a fixed dose of norepinephrine in septic shock [27]. In our observational study we considered a fixed step of 0.03 mcg/Kg/min for reduction of vasopressor dose and cannot exclude that a bigger reduction step may have affected venous return, thus influencing the relationship between EaDyn and MAP, despite the fact that some studies have raised concerns about this relationship [28]. The vasomotor tone is composed by vascular resistance and compliance and an increase in vasomotor tone affects both CO and SV [29]. Although norepinephrine acts as an α-agonist agent, its β1-agonist effects cannot be ignored [30] contributing, at low dosage, to preserve ventricular–arterial coupling, contrary to those agents only acting as α-agonists [31,32]. In consideration of this, a variation in CO due to the reduction in β1 stimulation, as well as the reduction in venous return, could affect MAP. Guarracino et al. [33] explored the relationship between ventricular–arterial coupling, MAP and norepinephrine variation finding an EaDyn > 0.83 as cut-off between responders and non-responders in MAP to norepinephrine increase; our data are consistent with these considering the reduction of drug dose. In our study, the multivariable logistic regression highlighted a statistically significant correlation between CI at T0 and MAP decrease. In the data shown by Guinot [11], the mean CO was higher than the CO observed in our study; therefore, the role of CO on MAP could have been negligible. In patients with a lower CI, the influence of CI should be considered in predicting variations on MAP [33].

In our cohort SVRI were not different between responders and non-responders and did not predict any change in MAP. This could be explained considering that SVRI only represents a component of vascular tone. Moreover, the energy stored in the arterial vessel wall after each heartbeat is not considered in SVRI measurement. EaDyn better represents the ventricular–arterial coupling, highlighting the concept that for each given SV a part of energy is transferred to the arterial wall and then released to maintain a continuous flow and contribute to the value of diastolic pressure [34]. If the arterial system is too compliant, a vasopressor infusion is needed to maintain the perfusion pressure. On the other hand, high levels of norepinephrine could impair left ventricular function, probably due to an increase of reflected waves, as demonstrated in an animal model [35]. For these reasons, the availability of a haemodynamic parameter which can be real-time evaluated, and that reflects the interaction between the heart and the vessels, could be of great interest to be used as a guide for vasopressors reduction and for norepinephrine infusion time minimization.

Considering the complexity of the haemodynamic regulation of a patient affected by septic shock, we argued that a single parameter as EaDyn could not be sufficient to fully predict the response to a norepinephrine decrease. In recent years, artificial intelligence has been involved in developing some complex algorithms to better describe patients at risk of suffering from haemodynamic instability [36], but the majority of studies have been targeted to the escalating phase of organ support and not to the de-resuscitation phase. The minimization of the human factor in the weaning phase of septic shock may be one of the answers in shortening the administration of vasoactive drugs when these are no longer needed. One randomized clinical trial compared clinical management to a closed-loop system for reduction of norepinephrine, with a significantly shorter administration time in the automatic group [9], even if this study only considered mean arterial pressure to decrease the norepinephrine infusion rate. The availability of tools that take into consideration multiple haemodynamic parameters may support the clinicians in making the right decision. For this reason, we developed a model based on the multivariate logistic analysis which could help to detect the probability of a MAP decrease greater than 10%. The derived score could be an interesting, easy-to-use tool to guide norepinephrine titration in septic-shock patients. However, a larger study is needed to validate this score. 

We excluded from this study patients who were on spontaneous breathing ventilation and those on mechanically controlled ventilation with tidal volume < 7 mL/Kg. PPV and SVV have been validated on positive pressure ventilation with a tidal volume > 7 mL Kg showing high sensitivity and specificity in predicting an increase of CO or SV due to volume expansion; meanwhile, their usefulness is limited in patients undergoing protective ventilation [37]. However, PPV and SVV can change differently during the respiratory cycle even if the predictive threshold for fluid responsiveness is not overcome due to the limited tidal volume. EaDyn represents the ratio between the two parameters and, as their relationship could potentially remain unchanged at lower values, EaDyn could be useful even in a protective ventilation setting or, as shown by Cecconi et al. [38], in a cohort of spontaneously breathing patients. The design of our study was intended to consider only tidal volume > 7 mL/Kg because of the lack of data to confirm the potentially good EaDyn performance on lower tidal volume ventilated patients. 

This study has several limitations. First of all, this is a single-centre study with a small sample size; moreover, as this is an observational study, large interventional studies are needed to confirm the clinical role of EaDyn as a guide to titrating norepinephrine dose. Furthermore, we considered only patients with infusion of norepinephrine without any other ongoing vasoactive drug; thus, our results cannot be taken into consideration in all septic patients. Moreover, we only considered one fixed step of norepinephrine reduction during our observation time; thus, we cannot draw conclusions about subsequent steps or different reduction doses and the subsequent EaDyn changes. Lastly, the threshold values of EaDyn to predict the fall in MAP > 10% after reduction of norepinephrine found in this study has to be considered strictly linked to the MostCare monitoring system algorithm and not generalizable to other haemodynamic monitoring systems, despite the EaDyn values being close to those measured by other devices [11,12,13,38].

## 5. Conclusions

In a septic-shock population, EaDyn at the steady state correlates well with a decrease of MAP greater than 10% after norepinephrine dose reduction of 0.03 mcg/Kg/min and therefore could potentially become a method useful to improve the vasopressor weaning phase. A prediction model based on EaDyn and other haemodynamic variables seems to predict well the risk of MAP variation at the beginning of the de-escalation phase of vasopressor support, suggesting that a global evaluation integrating different aspects could better represent the haemodynamic status of our patients. 

## Figures and Tables

**Table 1:** Demographic and clinical characteristics of the enrolled patients and septic shock origin. Quantitative variables are reported as median (first–third quartile). Qualitative variables are reported as number (percentage).

Variable	Value
Age (y.o.)	57 (47–66)
Gender (F/M) (%)	10/22 (31/69)
BMI (Kg/m^2^)	26.1 (21.4–29.4)
BSA (m^2^)	1.9 (1.6–2.0)
SOFA score	10 (9–12)
Vt/PBW (mL/Kg)	7.5 (7.2–8.0)
Arterial hypertension (%)	38
Diabetes mellitus (%)	26
**Source of septic shock (%)**	
Pulmonary	23 (72)
Abdominal	6 (19)
Other	3 (9)

Quantitative variables are reported as median (first–third quartile). Qualitative variables are reported as number (percentage). Abbreviations: F, female; M, male; BMI, body mass index; BSA, body surface area; SOFA, sequential organ failure assessment; Vt, tidal volume; PBW, predicted body weight.

**Table 2:** Variations of haemodynamic parameters pre and post norepinephrine dosage reduction; variables are reported as median (first–third quartile).

	Pre	Post
**Heart rate (bpm)**		
Responders	94 (93–104)	92 (91–102)
Non-responders	78 (70–100)	78 (68–95)
**Systolic blood pressure (mmHg)**		
Responders	127 (117–137)	107 (101–114) *
Non-responders	127 (113–134)	125 (111–131)
**Mean arterial pressure (mmHg)**		
Responders	72 (71–85)	64 (63–74) *
Non-responders	79 (76–92)	79 (73–91)
**Systolic volume (mL)**		
Responders	46 (37–52)	33 (31–40)
Non-responders	60 (40–67)	58 (42–68)
**Cardiac output (L/min)**		
Responders	4.3 (3.8–4.8)	3.2 (3.1–3.7)
Non-responders	4.4 (3.9–4.7)	4.4 (4.0–4.5)
**Systemic vascular resistances indexed (dynes × s/cm^5^)**		
Responders	1407 (1300–1489)	1413 (1387–1423)
Non-responders	1316 (1277–1483)	1307 (1274–1422)
**Stroke volume variation (%)**		
Responders	18 (15–18)	13 (10–14)
Non-responders	10 (6–13)	8 (6–12)
**Pulse pressure variation (%)**		
Responders	6 (4–7)	9 (8–10) *
Non-responders	6 (5–11)	6 (5–11)
**dP/dt (mmHg/ms)**		
Responders	1.36 (1.19–1.39)	0.88 (0.82–0.95)
Non-responders	1.03 (1.01–1.06)	1.01 (0.98–1.08)
**Norepinephrine infusion velocity (mcg/Kg/min)**		
Responders	0.14 (0.1–0.2)	0.1 (0.06–0.16) *
Non-responders	0.14 (0.09–0.2)	0.1 (0.06–0.17) *

* = *p* < 0.05 in comparison between the two groups (responders vs. non-responders).

## Data Availability

Not applicable.

## Supplementary Materials

The following supporting information can be downloaded at: https://www.mdpi.com/article/10.3390/life13010028/s1, Table S1: The multivariate analysis considering systemic vascular resistances indexed (SVRIpre), cariac index (CIpre), arterial elastance (Eapre), dynamic elastance (PPVSVVpre) all before drug reduction; Figure S1: The internal validation of the nomogram model performed at 1000 repetitions boot.
