# Supplementary material for: Dynamic Arterial Elastance to Predict Mean Arterial Pressure Decrease after Reduction of Vasopressor in Septic Shock Patients
Source: Life (Basel). 2022 Dec 22;13(1):28. doi: 10.3390/life13010028 (PMC9862728; doi:10.3390/life13010028)

Table S1. The multivariate analysis considering systemic vascular resistances indexed (SVRIpre), cariac index (CIpre), arterial elastance (Eapre), dynamic elastance (PPVSVVpre) all before drug reduction.

Outcome	Odds ratio	Std. err.	z	P>|z|	[95% conf.	interval]
SVRIpre	.9987234	.0012728	-1.00	0.316	.9962318	1.001221
CIpre	.0084215	.0180381	-2.23	0.026	.0001265	.5605168
Eapre	.1792535	.2990451	-1.03	0.303	.0068144	4.715312
PPVSVVpre	.0008729	.0020176	-3.05	0.002	9.41e-06	.0810019
_cons	2.84e+09	2.72e+10	2.27	0.023	19.98376	4.05e+17

Figure S1. The internal validation of the nomogram model performed at 1000 repetitions boot.

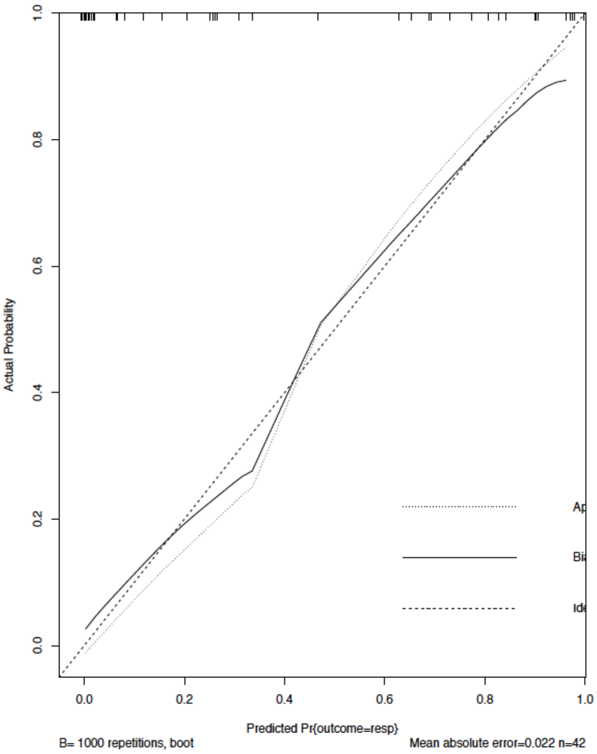

Supplement: Supplementary file 1 [file life-13-00028-s001.zip › life-2048291-supplementary.pdf]
